# Bilateral Ventral Pathways Support Phonological Awareness at Reading Onset in Spanish-Speaking Children

**DOI:** 10.1162/NOL.a.246

**Published:** 2026-04-23

**Authors:** Moramay Ramos-Flores, Rebeca Hernandez Soto, Liliana Sanchez-Zepeda, Fernando Lizcano-Cortés, Luis Concha, M. Florencia Assaneo

**Affiliations:** Instituto de Neurobiología, Universidad Nacional Autónoma de México, Querétaro, México

**Keywords:** phonological awareness, reading development, Spanish-speaking children, ventral and dorsal pathways, white matter tracts

## Abstract

Reading is a fundamental human skill that has been widely studied. While substantial progress has been made in identifying the white matter pathways supporting reading in adults, less is known about the neural substrates underlying reading acquisition in children. Moreover, existing evidence primarily focuses on a small set of languages, thereby raising questions about the generalizability of these findings. In this study, we address this gap by examining the white matter correlates of phonological awareness—a well-established precursor of reading development—in a cohort of monolingual Mexican Spanish–speaking children. Contrary to the classical view that left-lateralized dorsal pathways support phonological awareness, our results reveal that fractional anisotropy in bilateral ventral tracts, but not dorsal tracts, correlates with phonological awareness in this population. These findings challenge the traditional dichotomy between dorsal and ventral stream functions, instead highlighting the flexible and language-dependent nature of the neural mechanisms that support early reading development in children.

## INTRODUCTION

Reading is a complex cognitive skill that is intrinsically linked to the human experience and fundamental to daily life. Unlike other abilities that develop without explicit training, such as walking or speaking, reading is typically acquired during childhood through formal instruction. Over the past few decades, substantial effort has been devoted to understanding the core cognitive mechanisms and neural substrates that support reading in adults, resulting in a fairly comprehensive description (Coltheart, 2006; Dehaene, 2009; Dehaene et al., 2015).

However, researchers still debate how these mechanisms and their neural substrates evolve during development and how the specific features of the acquired language shape them. Existing findings cover only a limited number of languages, predominantly English (Lebel et al., 2019; Y. Wang et al., 2017; Yeatman et al., 2011), with some studies on Dutch (Vanderauwera et al., 2018), Chinese (Feng et al., 2020; N. Y.-H. Wang et al., 2021), and French (Dehaene-Lambertz et al., 2018; Feng et al., 2022). Recent studies using large data sets have included heterogeneous participants, but most of the data were collected in the United States (Carrión-Castillo et al., 2023; Roy et al., 2024). The underrepresentation of many languages in this field raises concerns about the generalizability of existing findings. In this study, we address this gap by assessing cognitive skills and white matter structure during the critical period when reading development begins to emerge, focusing on a Mexican Spanish–speaking population.

Evidence supports the existence of a neurocomputational model—known as the dual route cascaded model of reading (Coltheart et al., 2000, 2001)—when reading is established in adulthood or late infancy. This model proposes that reading is supported by two parallel routes. The phonological route is primarily used to read novel or infrequent words by mapping individual graphemes to their corresponding phonemes, whereas the orthographic or lexical route is used to read familiar words by decoding the entire letter pattern into the corresponding word. The dual-route model has been shown to correspond to specific white matter pathways, with a dorsal pathway supporting phonological processing and a ventral pathway underlying orthographic decoding, both lateralized to the left hemisphere (Vandermosten et al., 2012). A crucial question is whether left lateralization and the distinct functional roles of the dorsal and ventral streams are present from the early stages of reading acquisition or if they emerge later with experience and training.

In this context, several studies have investigated the brain structures underlying phonological awareness—a skill consistently identified as a precursor to reading acquisition across various languages (Carrillo, 1994; Hjetland et al., 2017; Hogan et al., 2005; Milankov et al., 2021; Sánchez & Escudero, 2017). Although there is broad agreement that phonological awareness is supported by the left dorsal pathway in both children (Lebel & Beaulieu, 2009; Perdue et al., 2025; Yeatman et al., 2011) and adults (Hickok & Poeppel, 2007; Saur et al., 2008) with established reading skills, the specific brain structures that underpin this ability before reading acquisition remain a matter of debate. The few studies conducted on children with prereading and early reading skills focus on a small set of languages and yield inconsistent results.

Two studies of American English–speaking children at prereading and early reading stages found that phonological awareness correlates with microstructural properties in the left dorsal pathway, specifically the left arcuate fasciculus (AF; Saygin et al., 2013) and the anterior segment of the superior longitudinal fasciculus (SLF; Travis et al., 2017). In line with these findings, longitudinal studies in native English speakers have identified properties of the left AF in infancy that are predictive of later phonological processing skills (Turesky et al., 2025; Zuk et al., 2021). In contrast, another work involving bilingual English-Spanish–speaking children found a correlation with the left inferior longitudinal fasciculus (ILF), a ventral stream tract, but no significant associations with dorsal pathways (Broce et al., 2019). Additionally, research on Canadian English–speaking children employing whole-brain analysis revealed associations between phonological awareness and microstructural properties in bilateral ventral pathways, specifically the bilateral inferior fronto-occipital fasciculus (IFOF), left uncinate fasciculus (UF), and right ILF (Walton et al., 2018).

Expanding on these findings, research with Dutch prereading children identified both bilateral ventral and left dorsal white matter pathways, namely, the bilateral IFOF and left AF, as a neural substrate supporting phonological awareness (Vandermosten et al., 2015). However, a more recent investigation examining the same language confirmed the relationship with the bilateral IFOF while demonstrating no significant associations with dorsal white matter pathways (Vandecruys et al., 2024).

Overall, experimental findings suggest that early in development—before children become proficient readers—the functional roles of the dorsal and ventral language streams are less fixed and significantly more dynamic.

In this context, it is crucial to investigate whether this developmental flexibility extends to Spanish-speaking pre and early readers, particularly because Spanish—unlike English and Dutch—has a transparent orthography characterized by a highly consistent phoneme–grapheme relationship (Carrillo, 1994; Defior, 2004; Jiménez González, 2002; Míguez-Álvarez et al., 2022; Rosselli et al., 2006; Ziegler & Goswami, 2005). In the present study, we evaluated a cohort of monolingual Mexican Spanish–speaking children with reading abilities ranging from prereading to early reading. Using a nuanced set of oral language assessments, we derived a composite score specifically indexing phonological awareness. Furthermore, we examined the microstructural properties of bilateral dorsal and ventral white matter pathways to investigate the neuroanatomical basis of phonological awareness—a cognitive precursor to reading—in this population.

## METHODS AND MATERIALS

This study was approved by the Ethics Committee of the Institute of Neurobiology, Universidad Nacional Autónoma de México (Protocol 096.H). All participants were informed about the task they were expected to complete. They provided their assent to participate, and their tutors gave informed consent.

### Participants

Sixty-one Mexican Spanish–speaking children (27 males, 34 females) between the ages of 6.0 and 8.5 years participated in the study. Participants were second- or third-grade students from a public elementary school in Queretaro, Mexico. The mean age was 7.3 years (*SD* = 0.6). The cohort consisted of 41 early readers and 20 prereaders, as determined by teacher reports and confirmed behaviorally using an oral reading task (see below). All children were monolingual Mexican Spanish speakers without neurological, developmental, or psychiatric disorders or contraindications for magnetic resonance imaging (MRI). Importantly, this cohort was assessed when children returned to school following the 2-year closures caused by the COVID-19 pandemic. This disruption to early formal education meant that participants were older than typically expected for the prereading and early reading stages, offering a unique opportunity to examine early literacy development in a delayed yet naturalistic context.

### Experimental Design

**Figure 1.**
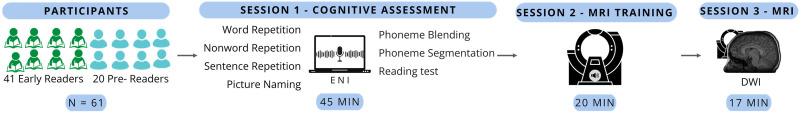
Overview of the experimental design. A total of 61 Mexican Spanish–speaking children (41 early readers, identified in green; 20 prereaders, identified in blue) participated in the three sessions. Session 1 consisted of a 45-min cognitive assessment, which included seven language-related tasks from the Neuropsychological Assessment of Children (*Evaluación Neuropsicológica Infantil*). Session 2 was a 20-min magnetic resonance imaging (MRI) training session to familiarize children with the scanning environment. Session 3 included a 17-min diffusion-weighted MRI. Sessions were conducted on separate days to minimize fatigue and optimize data quality.

Children were assessed in three sessions over a period of approximately 1 week (Figure 1). Session 1 took place at their school and involved a cognitive evaluation consisting of seven standardized language-related tasks, detailed in the next section. Session 2 took place on campus and included a 20-min MRI familiarization protocol. During this session, the children were exposed to an MRI simulator to help them become comfortable with the scanner environment. Session 3, also conducted on campus, involved the acquisition of diffusion-weighted MRI data. See below for a detailed description of each session.

### Cognitive Assessment (Session 1)

Language skills were assessed using seven tasks selected from the Neuropsychological Assessment of Children (*Evaluación Neuropsicológica Infantil* [ENI]), a standardized and validated cognitive battery for Latin American Spanish–speaking children (Matute et al., 2013). Tasks were administered individually using custom software built in MATLAB, with children seated in a quiet room, wearing headphones, and positioned close to a microphone. Each session lasted approximately 45 min per participant. All tasks were presented in the same fixed order across participants and were specifically chosen to be appropriate and accessible for both early readers and prereaders. All auditory speech stimuli were recorded by a professional female voice actor and digitized to ensure consistent presentation across participants. Below is a summary of each task:

*Word Repetition (seven items):* Participants listened to prerecorded words and were instructed to repeat each word aloud after its presentation. The task increased in difficulty, starting with one-syllable words and progressing to five-syllable words (syllable progression: 1-1-2-3-3-5-5). One point was awarded for each accurate repetition. Scoring errors included omissions, substitutions, or additions of phonemes.
*Nonword Repetition (eight items):* Participants listened to prerecorded pseudowords and were instructed to repeat each one aloud after its presentation. The task increased in complexity, beginning with single-syllable pseudowords and advancing to five-syllable items with greater phonological variability (syllable progression: 1-1-2-2-3-3-5-5). One point was awarded for each accurate repetition. Errors included phoneme omissions, substitutions, or additions.
*Sentence Repetition (nine items):* Participants listened to prerecorded sentences and were instructed to repeat each one aloud after its presentation. The sentences increased progressively in length and syntactic complexity, ranging from three to 17 words (word progression: 3-4-7-9-11-13-15-15-17). One point was awarded for each sentence repeated accurately, without semantic or phonological distortions.
*Picture Naming (15 items):* Participants were shown simple visual stimuli (e.g., a bird, a jacket) and instructed to name each item aloud. Responses were recorded, and 1 point was awarded for each correctly named item. Errors included incorrect labels or failure to respond.
*Phoneme Blending (10 items):* Participants listened to prerecorded sequences of isolated phonemes (e.g., /k/-/a/-/s/-/a/) and were instructed to blend them into a single spoken word (e.g., “casa”). Task difficulty increased progressively, starting with three-phoneme sequences and culminating in items with up to nine phonemes (phoneme progression: 3-4-4-4-6-6-7-7-9-9). One point was awarded for each correctly blended and articulated word.
*Phoneme Segmentation (10 items):* Participants listened to prerecorded words and, after each presentation, were asked to count the number of individual sounds (phonemes) in the word (e.g., “casa” → 4). Task difficulty increased progressively, ranging from three-phoneme words to words with up to eight phonemes (phoneme progression: 3-3-4-4-5-6-6-6-7-8). One point was awarded for each correct response, defined as accurately identifying and counting all phonemes in the word.
*Reading Aloud:* To identify early readers and prereaders in our cohort, all children were asked to read aloud a 101-word child-directed story. Participants were considered early readers if they could decode syllables and accurately read at least two complete words; children unable to decode syllables or read aloud at least two full words were considered prereaders. This information was used for descriptive purposes only, and no group-based analyses were conducted due to the imbalance between early (*n* = 41) and prereaders (*n* = 20).

### MRI Training (Session 2)

Before the MRI scanning session, participants completed a 20-min familiarization protocol designed to acclimate them to the scanning environment and improve compliance. During this session, participants remained in a mock scanner while being exposed to typical scanner noises. All participants successfully completed the protocol by remaining still for the entire session.

### MRI Acquisition (Session 3)

Participants were scanned on a 3T DISCOVERY MR750 scanner (General Electric Healthcare) equipped with a 32-channel head coil. High-resolution anatomical images were collected using a 3D axial FSPGR PURE T1-weighted sequence (TR/TE [Repetitions Time/Time Echo Time] = 7.048 ms/3.1 ms, TI [Inversion Time] = 450 ms, flip angle = 12°, slice thickness = 0.80 mm, voxel size = 1 × 1 × 0.8 mm^3^, FOV [Field of View] = 256 × 256 mm^2^, matrix = 256 × 256, 170 mm^3^). A 3D sagittal T2 CUBE sequence (TR/TE = 3,000/107.706 ms, flip angle = 90°, slice thickness = 1.0 mm, FOV = 220 × 220 mm^2^, matrix = 256 × 256, 160 mm^3^) was also acquired for anatomical reference.

Diffusion-weighted images were collected using spin-echo echo-planar imaging with a TR/TE of 1,000/80 ms, a resolution of 1 × 1 × 2 mm^3^, and a matrix of 128 × 128 × 62 mm, providing whole-brain coverage, which was then interpolated by the scanner to 256 × 256 × 62 mm using homodyne reconstruction. Diffusion sensitization was performed with two *b* values (800 and 2,500 s/mm^2^) applied along 32 and 64 noncollinear gradient directions, respectively. Five *b* = 0 s/mm^2^ were acquired before each shell, resulting in a total of 106 volumes. A reverse phase-encoding b0 pair was acquired in a separate run for susceptibility distortion correction. During image acquisition, children watched a movie of their choice and were accompanied by a parent or guardian to ensure comfort and minimize movement. Total acquisition time was approximately 13 min.

### MRI Preprocessing

Diffusion-weighted data were preprocessed using MRtrix3 Version 3.0.4 (Tournier et al., 2019) and FSL Version 6.0.7.14 (Jenkinson et al., 2012), following established guidelines. Noise was removed using a Marchenko–Pastur principal component analysis (PCA) approach implemented via the DESIGNER toolbox (Chen et al., 2024). To further improve image quality, Gibbs ringing artifacts were corrected using the mrdegibbs function in MRtrix3 (Kellner et al., 2016). This procedure is appropriate for our data, as the diffusion acquisition used full Fourier sampling. Finally, distortion and motion artifacts were corrected using FSL’s Eddy (Andersson & Sotiropoulos, 2016) run with CUDA acceleration. This step incorporated reverse phase-encoding images and phase-encoding parameters to achieve accurate geometric correction.

### Tractography and Tract Extraction

After preprocessing, automated fiber tracking and bundle segmentation were performed using the pyAFQ pipeline Version 2.1 (Kruper et al., 2021; Yeatman et al., 2012), following the workflow described in Tractometry (https://tractometry.org). Fiber orientation distributions were estimated in each voxel using constrained spherical deconvolution, as implemented in DIPY (Garyfallidis et al., 2014; Tournier et al., 2007), with an lmax of 8. Tractography was performed using a probabilistic approach with 1 million seeds per participant, generating streamlines within the white matter mask, and constrained using waypoint and endpoint regions of interest guided by morphing each subject’s native space into an anatomical template in Montreal Neurological Institute space. This procedure was employed to identify the following tracts bilaterally: SLF, ILF, IFOF, AF, and UF.

For each bundle, streamlines were iteratively cleaned across five rounds using Mahalanobis-based filtering to ensure anatomical specificity (Johnson et al., 2014). Each output was visually inspected to confirm segmentation accuracy and streamline plausibility. Tracts were resampled into 100 equidistant nodes per subject. Diffusion kurtosis fractional anisotropy (FA) was used to quantify white matter microstructure, capturing non-Gaussian diffusion properties. To ensure robust estimates of tract integrity, avoiding partial volume effects at the white/gray matter boundary and reducing the influence of crossing fibers near cortical endpoints, the mean FA value for each tract was computed by excluding the first and last five nodes (Kruper et al., 2021).

### Rotated PCA

Dimensionality reduction of the *Z*-scored cognitive data set was performed using PCA. Varimax rotation was employed to maximize the variance explained by each component and enhance interpretability, following the approaches described in previous studies (Kucukboyaci et al., 2014; Vanderauwera et al., 2018). Two components were retained based on a scree plot analysis (see Supplementary Figure S1; Supporting Information can be found at https://doi.org/10.1162/NOL.a.246). The PCA model explained 67% of the total variance, indicating that a small number of two components captured most of the structure in the data. These analyses were conducted using the “psych” package in R (Revelle, 2007). Principal component loadings on the original variables were normalized by dividing each loading by the maximum absolute value within its component.

### Statistical Analyses

Relationships between variables were assessed using Spearman’s rank-order correlations. Data points exceeding three standard deviations were excluded from the analyses. To control for Type I errors across multiple comparisons, all reported *p* values were adjusted using the false discovery rate (FDR) correction, with a significance threshold of *p* < 0.05.

## RESULTS

The main goal of the present study was to identify the white matter structures that support phonological awareness during the critical developmental window in which reading skills first emerge. To this end, we collected cognitive assessments and diffusion-weighted imaging data from a cohort of children at the early/prereading level (see Supplementary Table S1 for a detailed demographic description). We assessed participants' performance on six language-related tasks from the ENI (Matute et al., 2013), a cognitive battery designed for Latin American children: phoneme blending, phoneme segmentation, word repetition, nonword repetition, sentence repetition, and picture naming (see Supplementary Table S1 for a summary of performance on each task). Five bilateral white matter tracts associated with the dorsal (AF and SLF) and ventral (UF, ILF, and IFOF) pathways were selected as tracts of interest. While the left and right AF and the left IFOF were successfully traced in all participants, other tracts could not be reliably traced in a few participants (left SLF: *n* = 1; left ILF: *n* = 1; left UF: *n* = 5; right SLF: *n* = 1; right IFOF: *n* = 1; right ILF: *n* = 2; right UF: *n* = 3).

### Identification of Phonological Awareness Abilities

**Figure 2.**
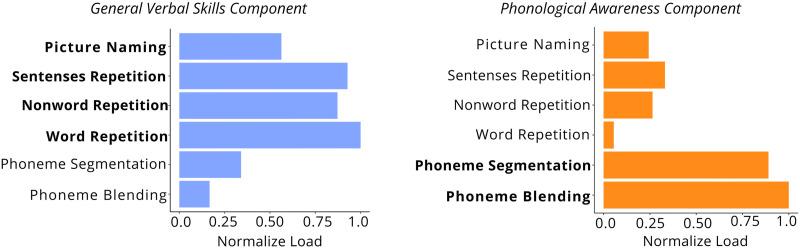
Cognitive components. Normalized loadings for the two principal components derived from the principal component analysis on the cognitive tasks. Left: The *general verbal skills component* (blue), primarily defined by high loadings on sentence repetition, word repetition, and nonword repetition. Right: The *phonological awareness component* (orange), with the highest loadings on phoneme blending and phoneme segmentation. Loadings were normalized within each component, with the most representative tasks highlighted in **bold**.

Rather than estimating phonological awareness skills using a predefined composite score of tasks, we adopted a data-driven approach aimed at achieving a more precise estimation of this ability. Specifically, we performed a rotated PCA (see Methods) on the resulting scores from the six evaluated tasks, aiming to identify a core dimension that represents phonological awareness. This analysis revealed that two components accounted for 67% of the total variance in the data. The first component, which explained 40% of the variance, exhibited high loadings on tasks such as word repetition, sentence repetition, and nonword repetition (see Figure 2 and Supplementary Table S2). Interpreting this component in detail is beyond the scope of the present study. However, it can be broadly defined as a *general verbal skills component* that encompassess speech production, working memory, and, to some extent, semantic processing. The second component, accounting for 27% of the variance, was primarily driven by phoneme segmentation and phoneme blending (see Figure 2 and Supplementary Table S2). Therefore, we interpret it as a *phonological awareness component* and will focus the following analyses on this specific measure. Importantly, the scores for this component showed no significant associations with the participants’ age, sex, or diffusion image quality (see Supplementary Figure S2).

### White Matter Structures Associated with Phonological Awareness Abilities

**Figure 3.**
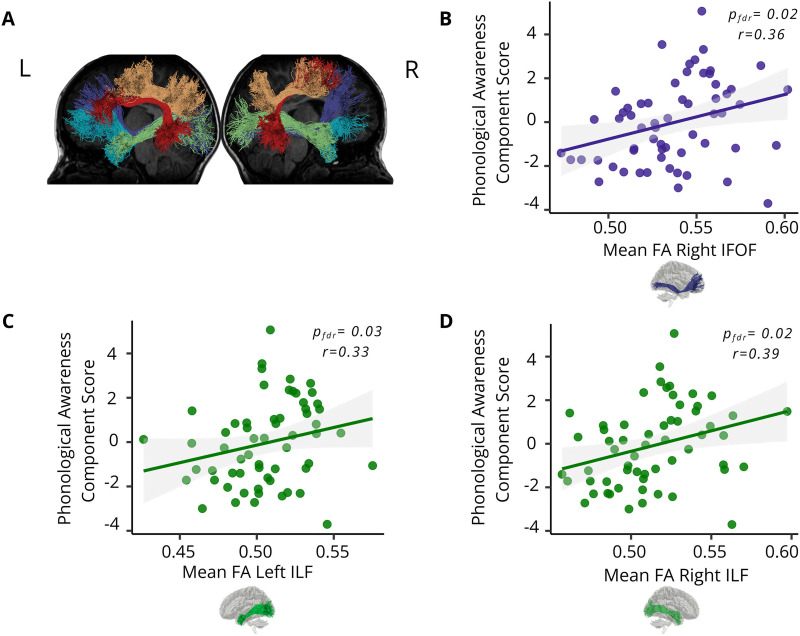
Significant correlations between neuroanatomical and cognitive measures. (A) Visualization of the 10 dissected white matter tracts in a representative participant, shown for both the left (top left) and right (top right) hemispheres: arcuate fasciculus (AF, red), superior longitudinal fasciculus (SLF, orange), uncinate fasciculus (UF, cyan), inferior fronto-occipital fasciculus (IFOF, violet), and inferior longitudinal fasciculus (ILF, green). (B–D) Scatter plots of phonological awareness component scores as a function of mean FA for three tracts: right IFOF (B), left ILF (C), and right ILF (D). In all panels, shaded areas represent 95% confidence intervals for the linear fits (solid lines), and dots represent individual participants.

This study focused on five bilateral white matter tracts (see Figure 3A), two of them belonging to the dorsal pathway (AF and SLF) and the remaining three to the ventral pathway (UF, ILF, and IFOF). For each tract, we computed the correlation between mean FA (see Methods) and the phonological awareness component score. The results showed no significant correlations between phonological awareness and the microstructural properties of the left or right dorsal pathways (right AF: *n* = 61, Spearman’s *r* = 0.10, *p*_fdr_ = 0.55; left AF: *n* = 61, *r* = 0.15, *p*_fdr_ = 0.34; right SLF: *n* = 60, *r* = 0.01, *p*_fdr_ = 0.93; left SLF: *n* = 60, *r* = −0.07, *p*_fdr_ = 0.63). In contrast, significant correlations were observed within the ventral stream, specifically for the right IFOF and the bilateral ILF (Figure 3B–D; right ILF: *n* = 59, *r* = 0.39, *p*_fdr_ = 0.02; left ILF: *n* = 60, *r* = 0.33, *p*_fdr_ = 0.03; right IFOF: *n* = 60, *r* = 0.36, *p*_fdr_ = 0.02; left IFOF: *n* = 61, *r* = 0.25, *p*_fdr_ = 0.10; right UF: *n* = 58, *r* = 0.25, *p*_fdr_ = 0.10; left UF: *n* = 56, *r* = 0.15, *p*_fdr_ = 0.34).

## DISCUSSION

The goal of this study was to identify the roles of dorsal and ventral white matter pathways supporting phonological awareness during the critical developmental period when children first acquire reading skills. Our findings highlight an association between bilateral ventral white matter pathways (particularly the right and left ILF and the right IFOF) and phonological awareness among Spanish-speaking children in the early stages of reading acquisition. Interestingly, no significant correlations were observed with the analyzed dorsal tracts.

As stated in the introduction, research on adults consistently identifies the left dorsal pathways as the primary structure supporting phonological awareness. However, findings in children are more heterogeneous. Developmental studies have reported differing results: Some identify exclusive involvement of the left dorsal pathway in phonological processing (Saygin et al., 2013; Zuk et al., 2021), while others report contributions from bilateral ventral pathways (Vandermosten et al., 2015). A third group of studies associates phonological processing with ventral tracts, revealing no significant involvement of the dorsal stream (Broce et al., 2019; Vandecruys et al., 2024). Notably, none of these studies have focused on monolingual Spanish-speaking children. The current study addresses this gap and contributes to the existing body of developmental neuroimaging research by underscoring the role of ventral white matter pathways in phonological processing among Spanish-speaking children.

It has been proposed that variability in previous findings may stem from differences in the age of the studied populations (Walton et al., 2018). During early development, the dorsal tracts are not yet fully mature, which can explain why phonological awareness is primarily supported by ventral pathways, which are already established. This function may therefore gradually shift to the left dorsal stream as structural maturation progresses (Brauer et al., 2013). Notably, the cohort in this study is older (6–8 years) than the typical age range reported in early reading studies (5–7 years). This age difference reflects the impact of the COVID-19 pandemic, which caused children in Mexico to miss about 1.5 years of in-person instruction, coinciding with the typical onset of formal reading education and thereby delaying it by roughly the same period. As a result, our sample includes older-than-usual early readers and prereaders, offering a unique opportunity to disentangle effects of developmental maturation from those of reading experience. Although the participants in this study are older and presumably have more mature dorsal pathways, our results show that phonological processing continues to correlate exclusively with ventral tracts. This finding challenges the idea that the dorsal stream inevitably assumes phonological functions once it matures. As an alternative hypothesis, we propose that the functional organization of these pathways may be influenced, at least in part, by the orthographic properties of the language being acquired. Further studies comparing matched samples of Spanish and English speakers would be needed to validate this hypothesis.

Interestingly, most studies highlighting a central role of the dorsal stream in phonological awareness have been conducted with English-speaking participants (Saygin et al., 2013; Travis et al., 2017). English has one of the most opaque orthographies, characterized by frequent irregular word mappings (Chomsky & Halle, 1968). In contrast, research on Dutch—a language with greater orthographic transparency than English (Borgwaldt et al., 2004)—more consistently reports the involvement of both dorsal and ventral pathways in phonological processing (Vanderauwera et al., 2018; Vandermosten et al., 2015). Our findings extend this line of research to Spanish, a language with a fully transparent orthography, where reading is posited to rely primarily on the phonological route (Rosselli et al., 2006). While reading in opaque orthographies requires the rapid creation and use of lexical representations, reading acquisition in transparent orthographies can rely exclusively on phoneme-to-grapheme mapping strategies (Kwok et al., 2017). In this context, lexical involvement is not essential during the early stages of reading acquisition for Spanish-speaking children, who have then both white matter routes (i.e., dorsal and ventral) available for phonological processing. Given this availability and the earlier maturation of the ventral stream, phonological processing at this stage is primarily supported by the ventral pathway. As reading becomes more automated and linguistic demands increase, lexical mechanisms are likely to become more engaged. It is plausible that when both phonological and lexical computations are required, phonological processing shifts to the left dorsal stream, while the ventral stream specializes in lexical integration. In line with this hypothesis, even in adulthood, the functional roles of the dorsal and ventral streams remain flexible and modulated by processing demands. For example, phonological word learning—a computation typically attributed to the left AF (López-Barroso et al., 2013)—can be supported by the ventral stream when the dorsal pathways are engaged in other concurrent tasks (Lopez-Barroso et al., 2011).

In summary, this study underscores the versatility of the brain’s white matter pathways and their functional organization during reading development. Our findings suggest that the involvement of the dorsal and ventral streams in phonological processing is not universally fixed but is instead shaped by the specific features of the orthography being learned. Specifically, we demonstrate that in Spanish—a language with a transparent orthography—phonological decoding correlates with bilateral ventral pathways, even at an age when dorsal pathways are expected to be fully mature. These results challenge traditional assumptions about the universality of neurocognitive routes in reading acquisition, emphasizing the need to move beyond English-centric models. Investigating a broader range of languages is essential for building a more comprehensive and inclusive understanding of the neural mechanisms underlying reading development. Such cross-linguistic research not only enriches theoretical frameworks but also informs educational practices tailored to the unique cognitive demands of different languages.

## Acknowledgments

We thank the parents and children who participated in the study. We also thank Azalea Reyes Aguilar for their enlightening conversations and Jessica Norris for proofreading the manuscript. Moramay Ramos-Flores is a doctoral student of the Programa de Doctorado en Ciencias Biomédicas at Universidad Nacional Autónoma de México (UNAM) and has received a fellowship (No.1061192) from Secretaría de Ciencias, Humanidades, Tecnología e Innovación (SECIHTI, formerly CONAHCYT).

## Funding Information

M. Florencia Assaneo, DGAPA-PAPIIT, Universidad Nacional Autónoma de México , Award ID: IN206825. M. Florencia Assaneo, Google (https://dx.doi.org/10.13039/100006785), Award ID: Gift 2021.

## Author Contributions

**Moramay Ramos-Flores:** Formal analysis: Lead; Investigation: Lead; Methodology: Equal; Validation: Lead; Visualization: Lead; Writing – original draft: Equal. **Rebeca Hernandez-Soto:** Data curation: Supporting; Supervision: Supporting; Writing – review & editing: Equal. **Liliana Sanchez-Zepeda:** Investigation: Supporting; Methodology: Supporting; Resources: Supporting. **Fernando Lizcano-Cortés:** Investigation: Supporting; Methodology: Supporting; Resources: Supporting. **Luis Concha:** Methodology: Supporting; Supervision: Supporting; Writing – review & editing: Equal. **M. Florencia Assaneo:** Conceptualization: Lead; Formal analysis: Supporting; Funding acquisition: Lead; Investigation: Supporting; Methodology: Equal; Project administration: Lead; Supervision: Lead; Visualization: Supporting; Writing – original draft: Equal.

## Code and Data Availability Statement

The preprocessed data and all analysis scripts are publicly available on https://openneuro.org/datasets/ds007398/versions/1.0.2 and https://github.com/moramay-rf/EarlyReading, respectively.

## Supplementary Material





## TECHNICAL TERMS

Phonological awareness: Ability to identify and manipulate phonemes, the basic sound units of spoken language.
Ventral and dorsal pathways: Two main white matter streams supporting language, each comprising multiple fiber bundles.
Diffusion-weighted imaging: A technique that allows to reconstruct white matter tracts and to estimate their microstructural properties.
Orthographic transparency: Degree to which graphemes (letters) consistently map onto phonemes (speech sounds) in a writing system.
