# Supplementary material for: Bilateral Ventral Pathways Support Phonological Awareness at Reading Onset in Spanish-Speaking Children
Source: Neurobiol Lang (Camb). 2026 Apr 23;7:NOL.a.246. doi: 10.1162/NOL.a.246 (PMC13137884; doi:10.1162/NOL.a.246)
Supplement: Supplementary file 1 [file nol-07-246-s001.pdf]

Bilateral ventral pathways support phonological awareness at reading onset in Spanish-speaking children

Moramay Ramos-Flores[1], Rebeca Hernandez Soto[1], Liliana Sanchez-Zepeda[1], Fernando Lizcano-Cortés[1], Luis Concha[1], M. Florencia Assaneo[1]*

[1] Instituto de Neurobiología, Universidad Nacional Autónoma de México; Querétaro, 76230, México.

* Corresponding author: M. Florencia Assaneo (fassaneo@inb.unam.mx)

Demographics			
Sex (male / fem)	27 / 34		
Grade (2nd /3rd)	40 / 21		
Age (mean +/- SD in years)	7.33 +/- 0.59		
Reading stage (early / pre readers)	41 / 20		
Cognitive assessment performance			
**Task**	**Mean # correct items (SD)**	**Range**	**Total items**
Picture Naming	14.16 (1.17)	12- 15	15
Sentence Repetition	2.88 (1.45)	0 - 6	8
Word Repetition	5.44 (1.37)	1 - 7	7
Non Word Repetition	6.27 (1.57)	0 -8	8
Phoneme Blending	1.41 (1.77)	0 - 8	10
Phoneme Segmentation	4.55 (3.07)	0 - 10	10

Supplementary Table 1. Demographic characteristics and cognitive assessment performance. Group distributions for sex, school grade, age and reading stage are reported. Mean scores, standard deviations (SD), score ranges, and total items are reported for each task.

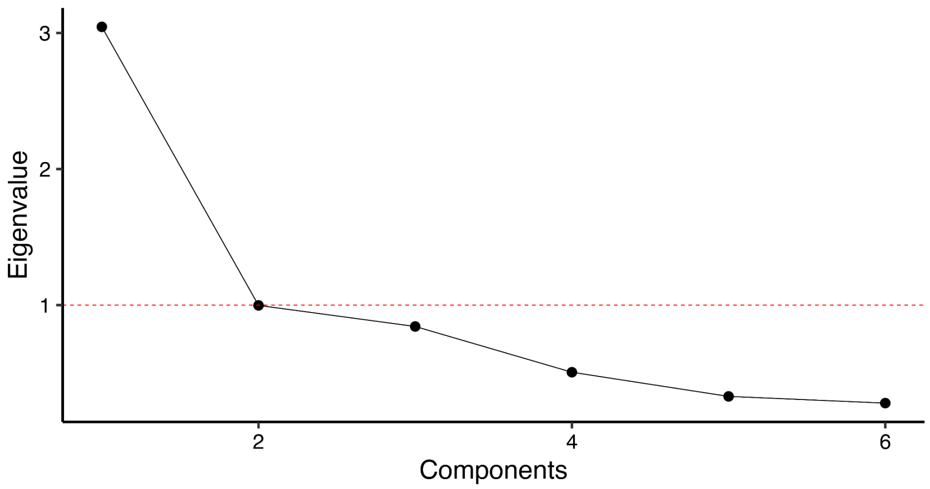

Supplementary Figure 1. Scree plot from the conducted PCA showing eigenvalues as a function of component number. The dashed line indicates the threshold of 1, commonly used as a criterion for selecting the number of components to retain.

COMPONENT LOADINGS (COGNITIVE)		
Task	*General Verbal Component*	*Phonological Awareness Component*
Phoneme Blending	0.15	0.90
Phoneme Segmentation	0.30	0.80
Non Word Repetition	0.77	0.24
Word Repetition	0.88	0.05
Sentence Repetition	0.82	0.30
Picture Naming	0.50	0.22

Supplementary Table 2. Rotated component loadings for each of the evaluated cognitive tasks. Loadings are derived from Principal Component Analysis (PCA) and reflect the contribution of each task to the identified cognitive components.

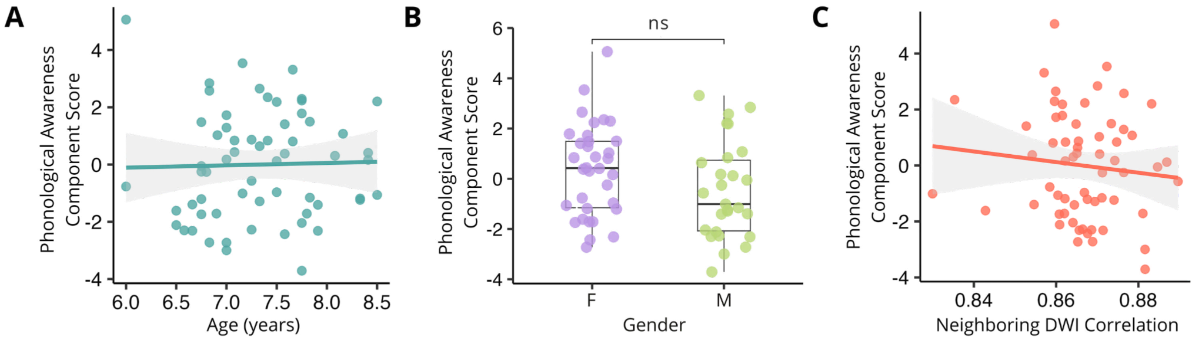

Supplementary Figure 2. Associations between phonological awareness scores and control variables. A. Phonological awareness scores did not correlate with age (Spearman's $r = 0.01$, $p = 0.39$). **B**. No statistically significant difference was observed between males and females (Wilcoxon rank-sum, $p = 0.09$). **C.** Similarly, no meaningful association emerged between phonological awareness scores and diffusion image quality (Spearman's $r = 0.02$, $p = 0.29$). Image quality was assessed using the Neighboring DWI Correlation (Yeh et al., 2019).

References

Yeh, F.-C., Zaydan, I. M., Suski, V. R., Lacomis, D., Richardson, R. M., Maroon, J. C., & Barrios-Martinez, J. (2019). Differential tractography as a track-based biomarker for neuronal injury. *NeuroImage*, *202*, 116131. https://doi.org/10.1016/j.neuroimage.2019.116131
